# Do Parental and Peer Support Protect Adjustment in the Face of Ethnic Discrimination? A Comparison between Refugee Youth and Youth of Immigrant Descent

**DOI:** 10.3390/ijerph182212016

**Published:** 2021-11-16

**Authors:** Julia Marie Christina Wenzing, Nadya Gharaei, Zeynep Demir, Maja Katharina Schachner

**Affiliations:** 1 Department of Educational Psychology—Culture & Socialization, Martin-Luther-University of Halle-Wittenberg, 06099 Halle, Germany; nadya.gharaei@paedagogik.uni-halle.de (N.G.); maja.schachner@paedagogik.uni-halle.de (M.K.S.); 2 Institute for Interdisciplinary Research on Conflict and Violence, Bielefeld University, 33615 Bielefeld, Germany; zeynep.demir@uni-bielefeld.de

**Keywords:** perceived ethnic discrimination, positive adjustment, refugee youth, social support, youth of immigrant descent

## Abstract

Applying a risk and protection perspective, this study paid special attention to the protective roles of parental and peer support in the face of perceived ethnic discrimination (PED) at school. Responding to the inconsistent findings of previous research, the survey study provides greater clarity regarding the interactions between PED at school, social support and positive adjustment (self-esteem, self-efficacy, optimism and school integration). The sample comprised 104 ethnic-minority youth (*M_age_* = 17.73, *SD* = 3.29, 61% female), including refugee youth (*n* = 55) and second- and third-generation youth of immigrant descent (*n* = 49). Structural equation models across the whole sample confirmed peer support as a significant moderator, indicating that ethnic-minority youth who received low peer support were less optimistic when facing PED. In multi-group models, we tested whether results differ across refugee youth and youth of immigrant descent. Results revealed between-group differences concerning the moderating roles of parental and peer support: For youth of immigrant descent, while more PED was associated with lower self-esteem when receiving low parental support, we found a positive association between PED and optimism when receiving high parental support. Based on the findings that refugee youth were shown to be less optimistic when obtaining low peer support, the main interaction effect for peer support on optimism seemed to be driven by refugee youth. The results of our cross-sectional study highlight the importance of identifying specific social support factors for specific adjustment outcomes and also the importance of differentiating between minority groups. Further, the findings offer practical implications for the educational sector in terms of programs focusing on the development of peer-support networks to especially promote refugee youth resilience and resettlement in Germany.

## 1. Introduction

European societies are heavily characterized by migration and cultural diversity. In 2015, more than 240 million international migrants were recorded, approximating 3.3% of the world’s population. From 2015 to 2017, Europe and Germany have witnessed the largest immigration movement in their recent recorded history [1]. According to the Federal Office for Migration and Refugees [2], approximately 500,000 asylum applications were submitted in Germany in 2015. Almost a third of these applications were submitted by youth under the age of 20 [2]. Next to refugee youth, youth of immigrant descent represent a large proportion of Germany’s population. They are defined as those who have at least one foreign-born parent or grandparent and either have been born outside (first generation) or within (second and third generation) Germany [3]. As the Federal Government Expert Commission [4] has recommended to use the term “immigrants and their direct descendants” to aggregate immigrants of second and later generations, we use the term “youth of immigrant descent” to describe the youth in our sample, who are descents of immigrants. For youth, who have recently migrated to and sought refuge in Germany, we use the term “refugee youth”. When applying the term “ethnic-minority youth” in our study, we encompass both refugee youth and youth of immigrant descent. Together, refugee youth and youth of immigrant descent aged under 20 cover more than one-third of the age-matched population in Germany [5]. It is thus clearly in the best interest of the German society to promote the positive adjustment of these young immigrant populations.

Similar to their non-immigrant descent peers, ethnic-minority youth (i.e., youth who are minoritized due to their ethnicity) face numerous developmental challenges, such as forming relationships with friends and family, a cohesive and secure sense of identity and developing responsibility [3,6]. In contrast to their non-immigrant descent peers, they further have to cope with significant acculturative challenges [7,8,9]. Acculturation can be described as a dynamic process through which individuals and groups from different cultures who are engaging in sustained contact adapt to one another [10]. For ethnic-minority youth specifically, salient stressors can arise from the acculturation process [11]. In this study, we focused on perceived ethnic discrimination (PED) as a major acculturative hassle which has been found in several studies to have detrimental consequences for the adjustment of ethnic-minority youth [12,13,14]. PED can be defined as the experience of being treated negatively based on one’s ethnic background [15] and has been identified as a powerful predictor of minority youth maladjustment [16,17,18]. The adjustment of immigrants and their descendants can be structured as encompassing both psychological and sociocultural outcomes [19,20]. While psychological adjustment captures their general well-being and mental health, sociocultural adjustment addresses cultural skills and functionally adaptive behaviors [21,22].

As a safe school environment with positive teacher and peer relations has substantial influence on youth development [23] and school forms one of the most important acculturative contexts for minority youth, we focus on PED at school by teachers and peers. Although there has been already research on PED in relation to adjustment for ethnic-minority youth [16,24], there is still little research specifically on refugee youth. Given this insufficient research and the rising numbers of refugee youth in Germany, we are particularly interested in possible differences between refugee youth and youth of immigrant descent in our study.

When studying the influence of PED on adolescents’ psychological development, the work on risk and protective factors offers a useful framework [25]. While risk factors can be defined as “individual or environmental hazards that increase an individual’s vulnerability for negative developmental behaviors, events, or outcomes” [26], protective factors serve as buffers so that the relations between risks and negative developmental outcomes are mitigated [25,27,28]. Thus, beyond viewing PED as a risk factor, this perspective considers factors that can protect the positive development of ethnic-minority youth in the face of PED. In the past, studies on the effects of ethnic discrimination have been criticized for focusing mainly on maladjustment and deficit environments [16]. In response to this criticism, the model of developmental risk and protective factors addresses psychological and environmental protective factors that can buffer against negative adjustment outcomes related to PED.

In this study, we specifically explore the role of PED at school by teachers and peers on ethnic-minority youth positive adjustment (in terms of their self-esteem, self-efficacy, optimism and school integration) while considering peer and parental support as possible protective factors. It extends previous research [29,30,31,32] in five ways, namely by (a) specifically examining the role of PED in the context of school instead of focusing on the general living environment of minority youth in Germany; (b) focusing on different aspects of positive adjustment (self-esteem, self-efficacy, optimism and school integration); (c) including three psychological (self-esteem, self-efficacy and optimism) and one sociocultural (school integration) indicator of adjustment, while most studies with immigrants only focus on psychological outcomes [33]; (d) considering the distinct roles of two key relational contexts by simultaneously testing parental and peer support as buffering factors concerning the relation between PED and adjustment; and (e) by including both refugee youth and second- and third-generation youth of immigrant descent, thus allowing us to explore potential differences in the risk and protective roles of PED and social support for their adjustment.

### 1.1. Perceived Ethnic Discrimination as a Risk Factor for Positive Adjustment

Being in an unsupportive and rejecting environment where individuals do not feel a sense of acceptance and relatedness, has been found to be a serious risk factor for adjustment [28]. Studies have shown that youth who are teased or called names by their peers at school have a higher chance to have a lower self-esteem and to do poorly in school [34,35]. In addition, adolescents who feel that their teachers do not respect or care about them, were identified to show more negative socioemotional and academic outcomes [36,37]. Overall, these messages of devaluation by teachers and peers can lead to negative developmental outcomes. Similarly, minority adolescents’ experiences of ethnic discrimination at school by teachers and peers can evoke the feeling of being devaluated because of one’s ethnic group membership. Thus, and as stated by Suárez-Orozco and colleagues [3] in their risk-and-resilience model, PED can act as a risk factor for minority youth, increasing the probability of negative developmental outcomes. In their meta-analytic study, Benner et al. [16] showed the pernicious effects of PED for adolescents on different indicators of well-being, such as poorer self-esteem, more depressive symptoms, greater psychological distress and lower academic achievements. In addition, Paradies et al. [38] and Priest et al. [24] highlighted PED as an important predictor negatively affecting ethnic-minority youth adjustment. In the current study, we specifically focus on PED at school by teachers and peers. Drawing on social identity theory, the asymmetric power relationship between teachers and students can promote differential treatment across groups of students based on teachers’ own social-group membership in relation to their students [39]. Teachers can use this asymmetric power relationship either in a positive and supportive way, or in a negative and destructive way. Studies revealed that experiences with ethnic discrimination by teachers are related to lower self-esteem, a weaker sense of school belonging and a poorer mental health [25,40,41]. Greene and colleagues [42] found that also experiences of racial discrimination at school from one’s peers predict declines in mental health, including increases in depression and decreases in self-esteem and psychological resilience. These results accord with several meta-analyses [14,24,38], which all identified a strong association between PED and poor adjustment in ethnic-minority youth. Indeed, the experience of ethnic discrimination was associated with low levels of self-esteem, life satisfaction, sense of coherence and high levels of depressive symptoms and other forms of psychological distress [14,24,38]. Considering the potential threat of PED to the healthy development of minority youth, PED can be classified as a high-risk factor for minority youth positive adjustment [25,26]. Keeping in mind that most research on PED and possible adjustment outcomes has been conducted with ethnic-minority youth in the US, we are particularly interested in studying these associations amongst refugee youth and youth of immigrant descent in Germany. In light of the past findings, we expect that PED at school decreases positive adjustment (in forms of self-esteem, self-efficacy, optimism and school integration) amongst refugee youth and second- and third-generation youth of immigrant descent in our current study.

### 1.2. Two Potential Protective Factors: Parental and Peer Support

While examining to what extent PED provides a risk for positive adjustment amongst ethnic-minority youth in Germany, it is necessary to identify factors than can buffer these effects. Within a risk and resilience framework in developmental psychology, protective factors can ensure that children and adolescents exhibit positive developmental outcomes despite at-risk contexts and challenges during adolescence [43]. Protective factors serve as buffers, ensuring that the relation between risks and problematic development is attenuated [27,28]. Several studies [44,45] showed that individuals with access to protective factors are likely to demonstrate more positive adjustment in comparison to those who do not.

There are a number of different strategies minority youth use to respond to ethnic discrimination [46]. In the present study, we examined whether social support acts as a protective factor while diminishing the detrimental effects of PED on the positive adjustment of ethnic-minority youth. When confronting PED, social support has been considered as an emotion-focused strategy [47] that indicates the availability of network members who express concern, love and care for the individual [48]. Several studies have shown that individuals are better able to cope with difficult circumstances when there is social support [49,50,51] and emphasized the role of social support as a facilitator for the positive adjustment of immigrants [52]. Further, the risk and resilience framework [50] emphasizes social support as an important protective factor for youth, while several studies identified social support as a commonly used coping strategy in the face of PED [53,54,55,56].

We specifically look at parental and peer support as two potential protective factors whose mitigating effects are understudied in the field of PED and as protective factors of ethnic-minority youth adjustment. In this study, we added to the literature by simultaneously testing the role of parental and peer support for the relationships between PED at school and multiple outcomes (self-esteem, self-efficacy, optimism and school-integration), as well as by studying these associations across two groups, namely refugee youth and second- and third-generation youth of immigrant descent.

The quality of the parent–child relationship is an important factor for the development of children and adolescents. Claiming that parents’ provision of consistent and responsive support leads to a sense of trust and competence in children, the Attachment Theory [57] emphasizes the importance of supportive parent–child relationships for children’s and adolescents’ emotional well-being. A number of studies have revealed that supportive parent–adolescent relationships characterized by secure attachments are related to positive psychological outcomes for youth, such as fewer mental health problems, lower levels of antisocial and aggressive behavior and more adaptive coping strategies [58]. Furthermore, parental support seems to have a similar beneficial effect on adjustment and health outcomes across racialized groups [30,59]. For example, the meta-analysis of Sun et al. [60] revealed that positive relationships between minority children of various racialized groups and their parents play a significant supportive role for their mental health. Better parent-child relationships were related to fewer mental-health problems and better mental-health outcomes.

Parental support has also been claimed as a protective factor for youth by fostering a closer bond between the parent and the child and consequently allowing for more open communication within the parent–child relationship and creating a sense of security for the child [57]. Indeed, studies of African American adolescents revealed supportive parental behavior as a buffer in the associations between perceived discrimination and behavior conduct problems and substance use [29,32]. Additionally, nurturing-involved parenting that includes emotional support, instrumental assistance and communication about potential areas of concern between parents and their children revealed to weaken the association between perceived discrimination and depressive symptoms. However, others have found that social-support networks do not moderate the perceived discrimination–mental health association [40].

Supportive peers may also serve as a protective factor against the negative effects of PED. Developmental considerations indicate an increased importance of peer support during adolescence [61]. As the relationship with one’s peers becomes increasingly important during adolescence, the protective effects of peer support seem to especially grow in importance in this developmental period [62]. Studies have found that supportive peer relationships increase the psychological well-being of ethnic-minority youth [31,63]. In addition, having a strong attachment with ethnic peers have been found to facilitate acculturation and enhance levels of well-being [64]. From an Attachment Theory perspective and especially for older ethnic-minority youth, close peers can provide alternative attachment relationships with similar benefits to parents [13]. Concerning the question whether peer support would moderate the relation between PED and positive adjustment outcomes, a stress-buffering model suggests that peer support would interact with PED to protect ethnic-minority youth from the negative effects of PED [65]. Regarding possible differences between refugee youth and youth of immigrant descent, peer support may act especially for refugee youth as a protective factor, as their parents often cannot support them because of mental strains and stress arising from the acculturation process.

Thus, in our study, we tested parental and peer support as possible buffers for both refugee youth and youth of immigrant descent. In addition, we had a closer look at possible similarities and differences between refugee youth and youth of immigrant descent, considering the insufficient research on PED and possible buffering effects especially in the case of refugee youth.

### 1.3. The Present Study

**Figure 1:**
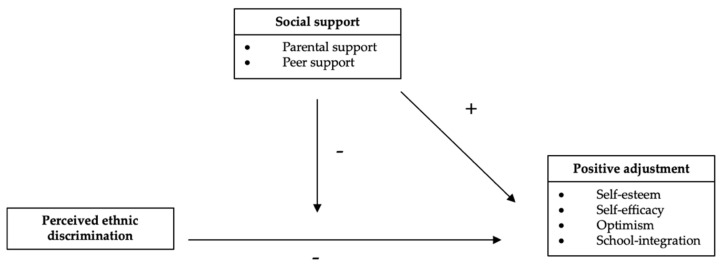
Summary of expected relations.

As shown in Figure 1, we investigated the associations of social support (parental and peer support) and different aspects of positive adjustment (self-esteem, self-efficacy, optimism and school integration) among ethnic-minority youth in Germany in the event of experiencing PED at school. Using a risk and resilience perspective, we expected that high experiences of ethnic discrimination at school would be associated with less self-esteem, self-efficacy and optimism for minority youth in Germany (Hypothesis 1a), but also with less school integration (Hypothesis 1b). Consistent with research showing that higher parental [30,58,59] and peer [31,63,64] support have beneficial effects on ethnic-minority youth mental health and adjustment, we, moreover, expected ethnic-minority youth to show high positive adjustment outcomes when receiving high parental (Hypothesis 2a) or peer support (Hypothesis 2b). Few studies have simultaneously tested parental and peer support as moderators for the association between PED and adjustment. Based on the risk and resilience perspective and some previous findings on the buffering effects of social support [66], it was hypothesized that parental (Hypothesis 3a) and peer (Hypothesis 3b) support will buffer the negative effects of PED on ethnic-minority youth self-esteem, self-efficacy, optimism and school integration. Finally, we also explored if our hypothesized model differed among refugee youth and second- and third-generation youth of immigrant descent (Exploratory Research Question 1).

## 2. Materials and Methods

### 2.1. Data and Participants

We used data from *N* = 104 ethnic-minority students in Germany, including *n* = 55 refugee youth (*M_age_* = 18.15, *SD* = 3.75, 49% female) and *n* = 49 youth of immigrant descent (*M_age_* = 17.27, *SD* = 2.64, 75% female). Data were collected in 2019 across various local contexts, including secondary schools (2), mosques (4), youth migration services (3), refugee shelters (1) and German language courses for refugees (3). Before starting data collection, the study received ethical approval from the ethics commission at Bielefeld University.

Secondary schools with high numbers of ethnic-minority students (>50%), along with mosques, youth migration services, refugee shelters and German language courses, were contacted personally and via phone. Prior to the administration of the paper-and-pencil questionnaire, informed consent from participants and their parents (for participants under the age of 18) was obtained. Participation was voluntary and not remunerated, anonymity was guaranteed and the participants were able to choose the language in which they wanted to answer the survey (German, Arabic or Kurmanji). Participants aged between 12 and 23 could take part in the survey.

The refugee status was based on the refugee definition presented in Article 1 of the United Nations Convention Relating to the Status of Refugees [67]. Consequently, adolescents who indicated in the questionnaire to have left their home country because of ethnic, religious and/or political persecution, discrimination, fear of violent conflicts and/or war were considered as refugees.

Refugee youth primarily stated Syria (*n* = 21), Turkey (*n* = 10) and Iraq (*n* = 7) as their countries of birth. Next to *n* = 45 youth of immigrant descent who specified to be born in Germany and to have at least one parent or grandparent who had immigrated to Germany (second and third generation), *n* = 4 indicated Turkey or Iran as their countries of birth. (Even though four participants indicated to be born outside of Germany, the vast majority of our respective subsample was indeed second- and third-generation youth of immigrant descent.) Ages of the total sample ranged from 12 to 23 (*M*_age_ = 17.73, *SD* = 3.29), and 61% identified themselves as female.

### 2.2. Measures

#### 2.2.1. Positive Adjustment

In this research, we assessed positive adjustment in terms of (a) four indicators of psychological well-being—namely self-esteem, two aspects of self-efficacy (relating to goals and abilities, respectively) and optimism—and (b) school integration. All positive adjustment outcomes were measured with items taken from the Developmental Resources Questionnaire for Children and Adolescents (FRKJ 8–16) by Lohaus and Nussbeck. Participants responded to the items on a scale ranging from 1 (*not at all*) to 4 (*all the time*).

The four indicators of psychological well-being were measured as follows. Self-esteem was assessed with three items: (1) “I can be proud of myself”, (2) “I feel good when I think about myself” and (3) “I have many good feelings when I think about myself” (α = 0.71). The two dimensions of self-efficacy were also assessed with a total of five items. The first dimension taps into how self-efficacious the ethnic-minority participants felt in reaching their goals: (1) “When I set a goal, I see the positive”, (2) “When I set my mind to something, I manage to do it”, and (3) “When I really want to achieve something, I also succeed” (α = 0.75). The second dimension captures self-efficacy in terms of abilities: (1) “With my skills I can achieve anything” and (2) “I can achieve a lot with my skills” (Spearman-Brown coefficient = 0.66). Optimism was assessed with a four-item scale, including the following items: (1) “I have a positive basic mood”, (2) “Even if I have problems, I see the positive”, (3) “If I am not doing so well, I know I will be better soon” and (4) “I believe that everything somehow turns out for the better” (α = 0.71). Finally, school integration was measured with six items: (1) “I feel comfortable at school”, (2) “I get along well with my classmates”, (3) “I really like the climate at my school”, (4) “All in all, I enjoy being together with my classmates”, (5) “My classmates are kind to me” and (6) “I feel comfortable in my class” (α = 0.88).

#### 2.2.2. Perceived Ethnic Discrimination at School

Perceived ethnic discrimination (PED) at school was assessed with five statements adapted from the Adolescent Discrimination Distress Index by Fisher, Wallace and Fenton [40]. First, the ethnic-minority adolescents read a short introduction: “After each statement, tell us if you have experienced each of the following types of discrimination because of your race or ethnicity. Remember we are only interested in occasions when racial/ethnic discrimination was at least partly responsible for your experience”. Afterwards, they were presented with five statements asking for a yes or no answer: (1) “You were discouraged from joining an advanced level class”, (2) “You were wrongly disciplined or given after-school detention”, (3) “You were given a lower grade than you deserved”, (4) “Peers did not include you in their activities” and (5) “People acted like they thought you were not intelligent”. As in previous research, the overall score was calculated by counting the items which were answered with a Yes answer.

#### 2.2.3. Social Support

To assess peer and parental support, we again used items from the Developmental Resources Questionnaire for Children and Adolescents (FRKJ 8–16) by Lohaus and Nussbeck; and ethnic-minority participants responded to the items on a scale ranging from 1 (*not at all*) to 4 (*all the time*). Peer and parental support were measured with six items each. The following items were used to measure peer support: (1) “I have friends I can rely on”, (2) “I also meet with friends after school”, (3) “I feel good when I am with my friends”, (4) “My friends like me as I am”, (5) “I think I am liked by others my age” and (6) “I have a lot of contact with my peers” (α = 0.76). For parental support, the youth answered the items (1) “If I need support, my parents are there for me”; (2) “If I am afraid of something, I can always approach my parents”; (3) “My parents are good at comforting me”; (4) “When I am in a bad mood, my parents take care of me”; (5) “I can always ask my parents for advice”; and (6) “I can rely on my parents” (α = 0.91).

#### 2.2.4. Control Variables

To estimate net effects of the main predictor variable, *age*, *gender* (1 = girls, 0 = boys) (while indicating their gender, participants could as well refer to the category “Other”; as only three participants selected this option, we recoded these three cases into missing values), *status* (1 = refugee youth, 0 = youth of immigrant descent), *school form* (1 = secondary education, 0 = post-secondary education) and *ethnic identification* were included as statistical controls in our models. As previous research [68,69,70,71] found significant links between our sociodemographic variables (age, gender, status and school form) and the well-being and school integration of ethnic-minority youth, we included them as control variables in our study. Further, several studies [25,72] found a significant link between ethnic identification and indicators of well-being and school adjustment for adolescents; therefore, we decided to include ethnic identification as a control variable. It was measured with the item “How strongly do you feel connected to your country of origin?” and for those who were born in Germany with the item “How strongly do you feel connected to the country of origin of your parents or grandparents?”. The ethnic-minority youth answered the question that applied to them on a scale from 1 (*not connected at all*) to 4 (*very strongly connected*).

### 2.3. Analyses

To test our hypotheses, we employed a structural equation modeling (SEM) procedure, using Mplus 8, version 1.6 [73]. In our model we controlled for status, age, gender, school form and ethnic identification. Concerning our additional analyses, multi-group analyses were performed with the aim of examining similarities and differences between refugee youth and youth of immigrant descent. Note that we do not have theoretical reasons to expect differences between refugee youth and youth of immigrant descent concerning the associations; however, we wanted to acknowledge these two distinct subgroups in our sample by testing whether our associations of interest are similar across the two subgroups. Three fit indices were considered to evaluate the model fit: comparative fit index (CFI > 0.90), Tucker–Lewis index (TLI > 0.90) and root-mean-square error of approximation (RMSEA ≤ 0.08) [74].

## 3. Results

### 3.1. Descriptive Results

Mean scores, standard deviations and ranges for our main study are presented in Table 1. Our participants reported a moderate number of experienced instances of PED at school (range: 0–5, *M* = 1.88, *SD* = 1.48). Concerning their psychological well-being, the mean scores of ethnic-minority youth self-esteem, self-efficacy and optimism were all close to the value 3 on their four-point scale, indicating psychological well-being on a rather high level. Similarly, school integration showed a high score of 3.01 (*SD* = 0.62). Finally, the mean scores of peer (*M* = 3.10, *SD* = 0.53) and parental support (*M* = 3.25, *SD* = 0.77) were close to the positive end of their four-point scale, suggesting rather high perceived support of parents and peers for ethnic-minority youth.

In addition, we report bivariate correlations between the main study variables in Table 2. Surprisingly, PED only showed a negative correlation with school integration but not with any other adjustment outcomes. Whereas both types of social support were associated with better school integration, more peer support was also associated with higher self-esteem, while more parental support was also associated with higher optimism. We also found a negative correlation between PED and parental support, while our adjustment outcomes were mostly positively related: significant positive correlations were found between self-esteem and self-efficacy, between self-esteem and optimism and between self-efficacy and optimism. In addition, better school integration was accompanied by higher self-esteem and optimism.

### 3.2. Main Analysis

#### 3.2.1. Perceived Ethnic Discrimination at School and Positive Adjustment Outcomes

We tested our hypotheses about the role of PED at school for ethnic-minority youth positive adjustment (self-esteem, self-efficacy, optimism and school integration) in a structural equation model. Model-fit indices indicate that this model fits our data well (CFI = 0.960, TLI = 0.919, RMSEA = 0.041). The results of our model, while controlling for status, age, gender, school form and ethnic identity, are presented in Table 3; unstandardized regression coefficients are reported.

We did not find significant relations between PED and minority youth psychological well-being: PED at school was unrelated to self-esteem, self-efficacy and optimism. Similarly, PED was unrelated to school integration. Thus, we have to reject both Hypotheses 1a and 1b: our findings do not provide evidence that minority youth who experience higher numbers of different instances of PED at school will show lower levels of psychological well-being (in terms of their self-esteem, self-efficacy and optimism) or school integration.

#### 3.2.2. Parental and Peer Support as Protective Factors

Regarding Hypotheses 2a and 2b, our model results show that both measures of social support were positively related to ethnic-minority youth psychological well-being and school integration. More precisely, ethnic-minority youth who received more parental support, were also more optimistic (*B* = 0.232, *p* = 0.009), felt more self-efficacious in terms of their abilities (*B* = 0.281, *p* = 0.003) and more integrated in school (*B* = 0.171, *p* = 0.046). For peer support, significant relations with self-efficacy (goals) (*B* = 0.299, *p* = 0.010), self-efficacy (abilities) (*B* = 0.276, *p* = 0.059), self-esteem (*B* = 0.477, *p* = 0.000) and with school integration (*B* = 0.266, *p* = 0.004) were found. Overall, these findings support Hypotheses 2a and 2b, namely that ethnic-minority youth who receive high amounts of parental and peer support will score higher on psychological well-being (in terms of self-esteem, self-efficacy and optimism) and school integration. However, as parental support was not significantly related to self-esteem and self-efficacy (goals) and minority youth who received more peer support were not significantly more optimistic, Hypotheses 2a and 2b were only partly confirmed.

Concerning Hypotheses 3a and 3b, there was a marginally significant two-way interaction effect between PED at school and peer support. To further examine this interaction, we conducted simple slope analysis [75].

**Figure 2:**
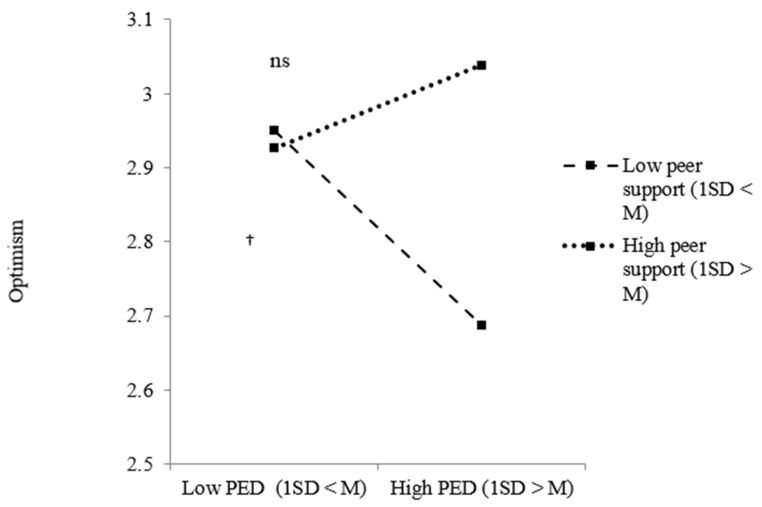
Effects of PED at school on ethnic-minority youth optimism dependent on the level of peer support. Note: ^†^ *p* < 0.10; ns = not significant.

We examined the effect of PED at school on minority youth optimism under two conditions: low (1 SD < M) and high (1 SD > M) peer support. As shown in Figure 2, ethnic-minority youth were less optimistic when they perceived ethnic discrimination, but only when there was low peer support (*B* = −0.092, *p* = 0.080). No significant effects were found when the amount of peer support was high. While Hypothesis 3b was partly confirmed, Hypothesis 3a—assuming that a (buffering) interaction effect between PED and parental support—could not be confirmed.

#### 3.2.3. Effects of Control Variables on Outcomes in Main Analyses

With regard to our control variables, we found that refugee youth were significantly more optimistic and reported more self-esteem and school integration than youth of immigrant descent. Moreover, compared to boys, girls revealed less self-efficacy concerning their abilities and less self-esteem. Age, ethnic identification and attending secondary vs. post-secondary education did not have a significant effect on ethnic-minority youth positive adjustment outcomes. As ethnic identification is known to be a significant protective factor in the face of PED [7,76], we also checked if our model results would change when leaving ethnic identification out as a control variable. Regarding this, our model results did not show significant changes.

### 3.3. Additional Analyses: Exploring Differences between Refugee Youth and Youth of Immigrant Descent

Although, we did not have theoretical reasons to expect differences between refugee youth and youth of immigrant descent concerning our model results, we still wanted to acknowledge these two subgroups in our sample and contribute to the closure of the aforementioned research gap. Therefore, we tested whether mean levels (Supplementary Materials Table S1) and associations are similar across the subgroups. Independent *t*-tests revealed that refugee youth showed better adjustment than youth of immigrant descent regarding their school integration, *t* (82.21) = −3.02, *p* = 0.003, *d* = −0.61, 95% CI [−0.60, −0.12]. Further, youth of immigrant descent reported significantly more types of experienced ethnic discrimination at school, *t* (102) = 2.68, *p* = 0.009, *d* = 0.53, 95% CI [0.20, 1.32]. Both significant differences embody medium to large-sized effects. For parental and peer support and our other adjustment outcomes no significant differences between refugee youth and youth of immigrant descent were revealed. In the next step, we tested for possible group differences in multi-group models that split our sample into the subgroups of refugee youth (*n* = 55) and youth of immigrant descent (*n* = 49). To ensure sufficient statistical power, we ran four separate multi-group models for self-esteem, optimism, the two self-efficacy measures (goals, abilities) and for school integration as independent variables respectively. The results of our multi-group models, while controlling for age, gender, school form and ethnic identity, are presented in Table 4.

Concerning the effect of PED at school on ethnic-minority youth psychological well-being and school integration, results indicated a significant positive effect of PED on refugee youth self-efficacy (aims) (*B* = 0.124, *p* = 0.081) and self-esteem (*B* = 0.210, *p* = 0.000) while for youth of immigrant descent no significant effects were found. Taking a closer look at our social-support measures, several differences between refugee and youth of immigrant descent were revealed: While for refugee youth no significant effects on optimism were found, for youth of immigrant descent a significant positive effect of parental support on optimism was revealed (*B* = 0.369, *p* = 0.002). Concerning our measures of self-efficacy, the results indicated significant effects for both refugee youth and youth of immigrant descent. For refugee youth, we found significant positive effects of parental (*B* = 0.294, *p* = 0.017) and peer support (*B* = 0.410, *p* = 0.040) on self-efficacy (abilities). For youth of immigrant descent, we also found a significant positive effect of parental support on self-efficacy (abilities) (*B* = 0.229, *p* = 0.059), as well as a positive effect of peer support on self-efficacy (goals) (*B* = 0.364, *p* = 0.020). Furthermore, our results presented significant positive effects of peer support on refugee youth (*B* = 0.760, *p* = 0.000) and youth of immigrant descents’ (*B* = 0.312, *p* = 0.015) self-esteem. Lastly, we found positive effects of peer support on refugee youth (*B* = 0.223, *p* = 0.048) and youth of immigrant descents’ (*B* = 0.251, *p* = 0.060) school integration. Additionally, parental support was positively related to school integration for refugee youth only (*B* = 0.264, *p* = 0.023).

**Figure 3:**
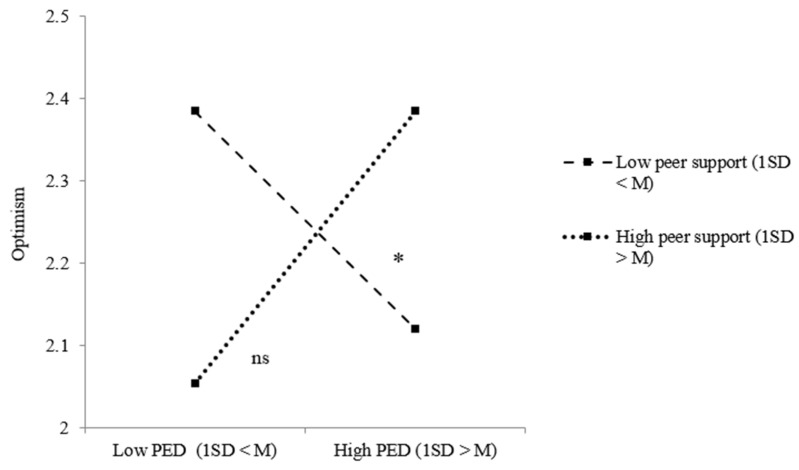
Effects of PED on refugee youth optimism dependent on the level of peer support. Note: * *p* < 0.05; ns = not significant.

**Figure 4:**
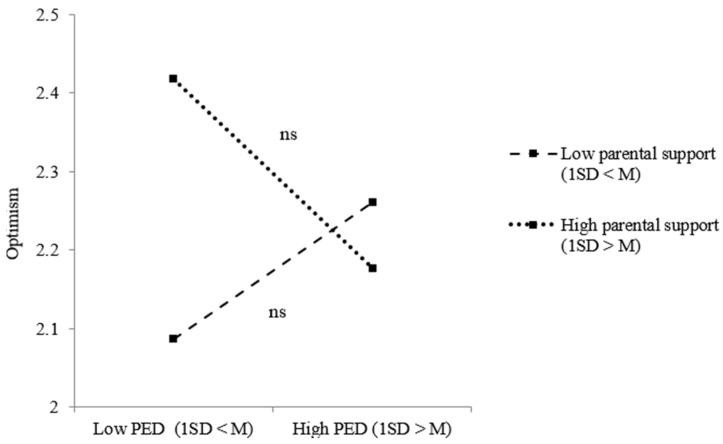
Effects of PED on refugee youth optimism dependent on the level of parental support. Note: ns = not significant.

**Figure 5:**
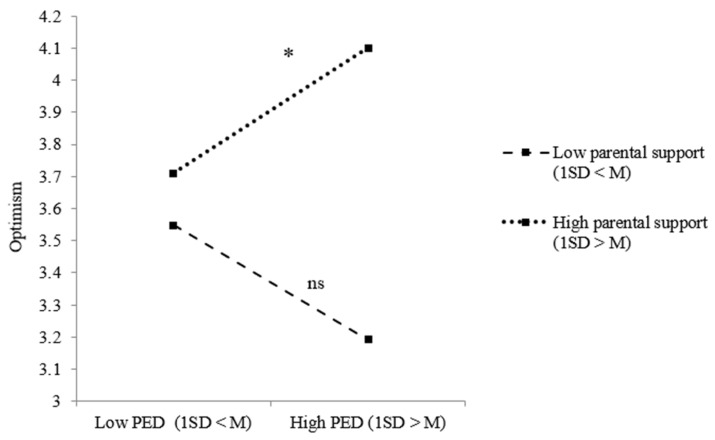
Effects of PED on youth of immigrant descents’ optimism dependent on the level of parental support. Note: * *p* < 0.05; ns = not significant.

**Figure 6:**
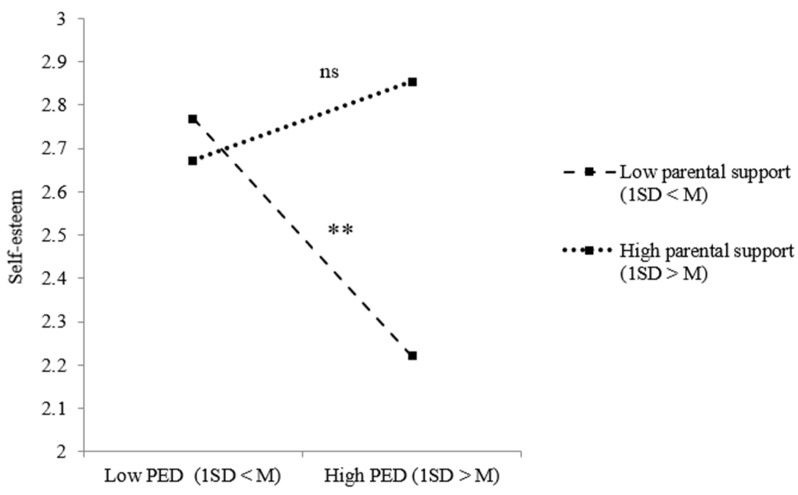
Effects of PED on youth of immigrant descents’ self-esteem dependent on the level of parental support. Note: ** *p* < 0.01; ns = not significant.

Taking a closer look at the relations between PED, social support and our positive adjustment measures, model results for refugee youth regarding optimism indicated both a significant two-way interaction effect between PED and peer support and between PED and parental support. Figure 3 shows that refugee youth were less optimistic when they perceived ethnic discrimination, but only when they received low peer support (*B* = −0.130, *p* = 0.037). Taking a closer look at the effect of PED on refugee youth optimism under low (1 SD < M) and high (1 SD > M) parental support, no significant effects were revealed (Figure 4). For youth of immigrant descent, significant two-way interaction effects between PED and parental support regarding optimism and self-esteem were revealed. Figure 5 shows the results of the simple slope analysis for youth of immigrant descents’ optimism: youth of immigrant descent were more optimistic when they perceived ethnic discrimination, but only when there was high parental support (*B* = 0.128, *p* = 0.043). As indicated in Figure 6, youth of immigrant descents’ self-esteem was negatively affected by PED when the amount of parental support was low (*B* = −0.180, *p* = 0.009).

## 4. Discussion

The research literature showing that greater PED is related to poorer adjustment for ethnic-minority youth [16,24,40,77] is growing. However, much less is known about potential protective factors that can buffer the detrimental effects of PED on ethnic-minority youth positive adjustment. The present study adds to the literature by simultaneously examining parental and peer support as buffering, protective factors concerning the potential negative relation between PED at school and adjustment. In addition, we explored possible differences between refugee youth and second- and third-generation youth of immigrant descent regarding the risk and protective roles of PED and social support for their adjustment.

Contrary to some previous research and our established hypotheses, we did not find that ethnic-minority youth show lower levels of adjustment in the form of lower self-esteem, self-efficacy, optimism or school-integration when experiencing higher numbers of different instances of PED at school. A possible explanation for this non-finding could be minority youth ethnic identification. Next to social support, ethnic identification is known to be another significant protective factor in the face of PED [7,76]. Referring to the rejection-identification model by Branscombe and colleagues [78], it may be that most of the minority youth highly identified with their ethnic group after they have experienced discrimination against their ethnic group, which protected them from the negative effects of PED. Future research is needed to examine our models, while also including ethnic identification as another protective factor. Although we did not find significant changes in our model results when leaving ethnic identification out as a control variable, future research should further examine ethnic identification as another protective factor.

Moreover, contrary to our expectation, we found in our additional analyses that refugee youth who experienced more instances of ethnic discrimination also reported higher self-esteem and more self-efficacy in achieving their goals. A possible explanation could be psychological reactance [79]. As stated in Brehm’s reactance theory [80], psychological reactance describes a motivational state caused by a perceived threat to an individual’s freedom. While Wareham and colleagues [79] have found experiences of discriminatory events to cause individuals to experience a reactive state, in our study refugee youth higher self-esteem and self-efficacy could be effects of their psychological reactance caused by high PED. The fact that we only observed a possible reactive effect amongst refugee youth may also be an indicator of higher levels of resilience compared to youth of immigrant descent. This may be a result of their flight experience and many challenges they had to face and overcome. Additional research is needed to examine in more detail the psychological mechanisms underlying the found associations.

Furthermore, our study results suggest that both parental and peer support are beneficial for ethnic-minority youth positive adjustment. As expected, and found in previous research [61,65], minority youth who experienced more parental or peer support also reported higher self-esteem, as well as greater self-efficacy, optimism and school integration. Moreover, similar overall patterns were found across refugee youth and youth of immigrant descent. Interestingly, parental support was positively associated with school integration only for refugee youth and positively associated with optimism only for youth of immigrant descent. This shows that parental support can affect refugee youth and youth of immigrant descent in different ways, thus highlighting the importance of distinguishing between the two groups. Future research should study the mechanisms that underlie and can explain these different associations across the two groups.

With regard to parental and peer support as protective factors in the face of PED, we found that ethnic-minority youth who experienced low peer support were less optimistic when experiencing PED. In contrast, optimism was unrelated to experiences of PED when peer support was high. Here our results examining associations across refugee youth and youth of immigrant descent suggest that this buffering effect of peer support is mainly driven by refugee youth. For youth of immigrant descent, we found that high parental support protected against negative effects of PED on self-esteem, while it may even have contributed to an overcompensation for optimism, which was not affected by PED in the first place. We propose that parental racial socialization may explain the sustained self-esteem and optimism of youth of immigrant descent, when experiencing PED.

Racial socialization by parents includes teaching their children about race and ethnicity, enhancing their sense of ethnic identity and preparing them for experiences of ethnic discrimination [81]. Previous research has shown that parental racial socialization can be associated with positive outcomes, for instance, in Black youth experiencing racial stress [82]. As many parents of youth of immigrant descent have already spent much time in Germany and have gone through processes of acculturation and possibly also experiences of ethnic discrimination themselves, they may be better equipped to racially socialize and empower their children than parents of newly immigrated refugee youth. This in turn may explain why youth of immigrant descent in our study were able to sustain their self-esteem and optimism in the face of PED when parental support was high. It would be interesting for further studies to examine parental support in the form of racial socialization in relation to PED and youth adjustment. Additionally, it may be interesting to explore if the socioeconomic conditions of the parents could explain the sustained self-esteem and optimism of youth of immigrant descent. Considering, that parents of youth of immigrant descent have a higher socioeconomic status (SES) than parents of refugee youth, they possibly have more resources to support their children. Future studies should therefore collect more data about the SES conditions of parents, including parental education and income.

Another interesting avenue for future research—especially in view of the rising numbers of refugees in Germany and also the rising numbers of racial attacks and racism—would be to examine how parents of refugee youth in Germany can be empowered to provide the kind of support that their children need in the face of PED. Taking into account that parents of refugee youth, similar to their children, are going through the process of acculturation and must cope with salient stressors arising from the acculturation process, refugee youth possibly seek more support from their peers to not put additional burden on their parents. For that reason, receiving support from external sources—such as from the school—in the process of acculturation is important for parents and needs to be further enhanced. While parents would be relieved, refugee youth would dare again to ask their parents for support and share their experiences of discrimination with them.

### 4.1. Limitations and Future Research

The present study should be considered in the light of some limitations and qualifications. First, we used cross-sectional data and therefore could not provide evidence for causality. We analyzed PED and parental and peer support as predictors of ethnic-minority youth positive adjustment, but there could be reciprocal influences. However, there are a handful of longitudinal studies, suggesting PED to have deleterious effects on youth adjustment [42,83]. Future studies should use longitudinal designs to replicate our findings. Additionally, future longitudinal studies are needed to test directional, longitudinal moderation enabling to understand to what extent family and peer processes offer protection against ethnic discrimination over time.

As a second limitation, we acknowledge that the present study used measures for PED that only captured the number of different instances of PED, but not their frequency and intensity in a given period of time. It could be the case that ethnic-minority youth experienced the same instance of PED at school multiple times and in different intensities. At the same time, youth may have responded to the items with different timeframes in mind, with some only thinking about their recent experiences and others thinking about all experiences they ever made. This may make responses on this scale less comparable across individuals and may be another reason for less associations of PED with other variables compared to studies using measures that are more standardized in terms of the timeframe they address. So, these flaws of the measure of PED may have contributed to an underestimation of PED effects in the given study. Future studies should use measurement instruments for PED, which also enable to make statements about the amount and intensity of PED and not only about the experienced number of instances of PED, and which state a specific time frame, such as experiences in the last month or year. Furthermore, our social-support measures were not specifically matched to the needs that might be elicited by PED. Future studies should therefore go beyond questions that ask about general peer or parental support to assess specific support needs when dealing with PED. Specific support needs may, for instance, be linked to parental racial socialization practices [82] and the roles of co-ethnic and other-ethnic peers [84]. As Juang et al. [13] recommended, one way forward could be, for instance, to study how parents and peers react when they are told about discrimination experiences.

### 4.2. Implications

The results of our study suggest several practical implications. As both parental and peer support were associated with ethnic-minority youth positive adjustment, and especially peer support was found to be a significant protective factor for refugee youth in Germany, implementing programs at schools to promote peer and parental support is of high importance.

Taking into account that positive intergroup contact has been found to reduce prejudices against outgroup members [85,86] and considering the positive effects of interethnic friendships for minority youth adjustment [87], supporting the interaction and experiences with peers of other racial groups represents a favorable recommendation for action in this context. Therefore, the early creation of opportunities for positive contact experiences, especially between refugee youth and non-refugee youth, is of central relevance. By giving the opportunity to break down prejudices and promoting the development of peer networks, joint sports activities and programs where ethnic-minority youth and youth from the majority society form a tandem have high potential to enhance ethnic-minority youth positive adjustment [88]. Having the aim to support schools in helping ethnic-minority youth to develop a positive ethnic identity and promoting their social integration by forming such tandems, programs as the one developed by Zander and colleagues [89] in Germany present suitable interventions for schools. In tandems, youth learn more about their own and others’ values and emotions and reflect on friendship and the changeability of established normed. It becomes clear that joint activities are paramount and need to be promoted by schools within and outside the classroom.

Referring to the significant role of parental support for ethnic-minority youth positive adjustment, a good cooperation between schools, teacher and parents is essential. Previous research has shown that such successful cooperation can have positive effects on youth development, academic outcomes and future opportunities [90]. In this context, it needs to be considered that, for parents of refugee youth who are generally very interested in their children’s school career and positive development, insufficient language skills constitute a major barrier [91]. Consequently, schools in Germany would do well in providing interpreters and cultural brokers, enabling parents to take part in parent nights and consultations and receiving and understanding the information they need to better support their children. Furthermore, the establishment of meeting places where parents can have a mutual exchange would make it possible for parents to create new contacts, to exchange about how to support their children best and, at the same time, also manage their own acculturation process [88]. Referring to the finding that refugee youth often are able to integrate more quickly than their parents and thus become cultural brokers for their parents [92], the provision of support for parents regarding their own acculturation process would be beneficial for refugee youth. While their parents would be relieved, refugee youth would no longer have the feeling to burden their parents and would dare to open up and seek parental support regarding their discrimination experiences. Thus, they could experience parental support as an important buffer for their discrimination experiences. It becomes clear that the strengthening of cooperation between schools and social community agencies is of central importance.

## 5. Conclusions

Ethnic-minority youth spend a considerable part of their waking hours at school while interacting with peers (classmates) and teachers. The relations between PED, parental and peer support and positive adjustment, as well as research on possible similarities and differences amongst refugee youth and youth of immigrant descent regarding these associations, have received little attention so far. The results of our study show that PED may hurt less for some youth, depending on their level of support by parents and peers. Further, our study provides information on the differences between refugee youth and youth of immigrant descent regarding their experiences of PED and needs for support in the German context. Future research should examine the mechanisms that underlie and can explain these different experiences and associations across the two groups. Moreover, schools and teachers in Germany would do well to implement programs that strengthen peer networks and the ability of parents to support their children, while also addressing the issue of ethnic discrimination at school.

## Figures and Tables

**Table 1:** Means and standard deviations of our main study variables (*N* = 104).

	*M*	*SD*
Self-esteem	2.80	0.63
Self-efficacy (goals)	2.89	0.66
Self-efficacy (abilities)	3.01	0.65
Optimism	2.93	0.58
School integration	3.01	0.62
Perceived ethnic discrimination at school	1.88	1.48
Peer support	3.10	0.53
Parental support	3.25	0.77

Note: All variables except for PED were measured on a scale from 1 to 4. PED represents a count variable, counting types of experienced ethnic discrimination at school from 0 to 5.

**Table 2:** Correlations between main study variables (*N* = 104).

	1	2	3	4	5	6	7	8
Perceived ethnic discrimination at school	--							
2. Self-esteem	−0.001	--						
3. Self-efficacy (goals)	0.173 ^†^	0.440 ***	--					
4. Self-efficacy (abilities)	0.062	0.428 ***	0.377 ***	--				
5. Optimism	−0.103	0.440 ***	0.409 ***	0.384 ***	--			
6. School integration	−0.292 **	0.403 ***	0.023	0.187 ^†^	0.246 *	--		
7. Peer support	−0.008	0.274 **	0.159	0.133	0.089	0.320 ***	--	
8. Parental support	−0.339 ***	0.112	−0.030	0.164 ^†^	0.274 **	0.426 ***	0.232 *	--

Note: *** *p* < 0.001, ** *p* < 0.01, * *p* < 0.05 and ^†^ *p* < 0.10.

**Table 3:** Results of the structural equation model (*N* = 104).

	Self-Esteem	Optimism	Self-Efficacy (Goals)	Self-Efficacy (Abilities)	School Integration
Perceived ethnic discrimination at school	0.031	−0.026	0.063	0.039	−0.038
Peer support	0.477 ***	0.163	0.299 **	0.276 ^†^	0.266 **
Parental support	0.120	0.232 **	0.053	0.281 **	0.171 *
*2-way interaction terms*					
PED at school X Peer support	0	0.130 ^†^	0	0	--
PED at school X Parental support	0	0	0	0	--
*Controls*					
Status (Refugee)	0.262 *	0.237 *	0.039	0.060	0.307 **
Age	−0.031	−0.013	0.007	−0.036	−0.002
Gender (Female)	−0.219 ^†^	0.147	−0.065	−0.251 *	0.079
School form (Secondary school)	−0.066	−0.078	0.098	−0.093	−0.120
Ethnic identification	−0.180 **	0.021	−0.046	−0.020	−0.043
*Variance*					
R^2^	0.300	0.192	0.089	0.207	0.218
*Model fit*					
RMSEA	0.041				
CFI	0.960				
TLI	0.919				

Note: Unstandardized coefficients are presented; *** *p* < 0.001, ** *p* < 0.01, * *p* < 0.05 and ^†^ *p* < 0.10.

**Table 4:** Multi-group models comparing refugee youth (*n* = 55) and youth of immigrant descent (*n* = 49).

	Model 1	Model 2	Model 3		Model 4
	Self-Esteem	Optimism	Self-Efficacy (Goals)	Self-Efficacy (Abilities)	School Integration
**Refugee youth (*n* = 55)**	*B*	*B*	*B*	*B*	*B*
Perceived ethnic discrimination at school	0.210 ***	−0.013	0.124 ^†^	0.103	0.017
Peer support	0.760 ***	0.034	0.234	0.410 *	0.223 *
Parental support	−0.050	0.089	0.079	0.294 *	0.264 *
Perceived ethnic discrimination at school X Peer support	0	0.242 *	0	0	0
Perceived ethnic discrimination at school X Parental support	0	−0.117 *	0	0	0
**Youth of immigrant descent (*n* = 49)**					
Perceived ethnic discrimination at school	−0.060	0.006	0.012	0.032	−0.037
Peer support	0.312 *	0.096	0.364 *	0.048	0.251 ^†^
Parental support	0.185	0.369 *	0.026	0.229 ^†^	0.055
Perceived ethnic discrimination at school X Peer support	0	0	0	0	0
Perceived ethnic discrimination at school X Parental support	0.165 *	0.169 *	0	0	0

Note: Unstandardized regression coefficients are reported. For simplicity, control variables are not shown, included as controls in the model are age, gender, school form and ethnic identification; *** *p* < 0.001, * *p* < 0.05 and ^†^ *p* < 0.10.

## Data Availability

Not applicable.

## Supplementary Materials

The following are available online at https://www.mdpi.com/article/10.3390/ijerph182212016/s1. Table S1: Independent *t*-tests results comparing refugee youth (*n* = 55) and youth of immigrant descent (*n* = 49) on our main study variables.
