# Supplementary material for: Do Parental and Peer Support Protect Adjustment in the Face of Ethnic Discrimination? A Comparison between Refugee Youth and Youth of Immigrant Descent
Source: Int J Environ Res Public Health. 2021 Nov 16;18(22):12016. doi: 10.3390/ijerph182212016 (PMC8625724; doi:10.3390/ijerph182212016)
Supplement: Supplementary file 1 [file ijerph-18-12016-s001.zip › ijerph-1426046-supplementary.pdf]

Table S1. Independent t-tests results comparing refugee youth (n = 55) and youth of immigrant descent (n = 49) on our main study variables.

						95% CI		
	N	***M***	***SD***	***t***	***p***	***LL***	***UL***	**Cohen's *d***
**Perceived ethnic discrimination at school**								
Refugee youth	55	1.53	1.29	2.68	0.009 **	0.20	1.32	0.53
Youth of immigrant descent	49	2.29	1.60					
**Self-esteem**								
Refugee youth	55	2.90	0.66	−1.66	0.100	−0.45	0.40	−0.33
Youth of immigrant descent	49	2.69	0.58					
**Self-efficacy (goals)**								
Refugee youth	55	2.86	0.65	0.45	0.656	−0.20	0.31	0.09
Youth of immigrant descent	49	2.92	0.67					
**Self-efficacy (abilities)**								
Refugee youth	55	3.01	0.66	0.01	0.993	−0.25	0.25	0.01
Youth of immigrant descent	49	3.01	0.63					
**Optimism**								
Refugee youth	55	3.00	0.52	−1.22	0.226	−0.36	0.09	−0.24
Youth of immigrant descent	49	2.86	0.64					
**School integration**								
Refugee youth	55	3.18	0.47	−3.02	0.003 **	−0.60	−0.12	−0.61
Youth of immigrant descent	49	2.82	0.64					
**Peer support**								
Refugee youth	55	3.04	0.49	1.30	0.198	−0.07	0.34	0.26
Youth of immigrant descent	49	3.17	0.56					
**Parental support**								
Refugee youth	55	3.35	0.70	−1.40	0.163	−0.21	0.15	−0.28
Youth of immigrant descent	49	3.14	0.84					

Note. CI = confidence interval. *LL* = lower limit. *UL* = upper limit. All variables except for PED were measured on a scale from 1–4. PED represents a count variable, counting types of experienced ethnic discrimination at school from 0–5. $^{**}p < 0.01$.
